# The impact of cerebellar transcranial direct current stimulation on isometric bench press performance in trained athletes

**DOI:** 10.1016/j.heliyon.2024.e29951

**Published:** 2024-04-18

**Authors:** Rouven Kenville, Martina Clauß, Stefan Berkow, Patrick Ragert, Tom Maudrich

**Affiliations:** a Department of Movement Neuroscience, Faculty of Sports Science, Leipzig University, Leipzig, 04109, Germany; b Department of Neurology, Max Planck Institute for Human Cognitive and Brain Sciences, Leipzig, 04103, Germany

**Keywords:** Cerebellar tDCS, Bench press, EMG, Muscle activation, Maximum isometric force

## Abstract

Athletic development centers on optimizing performance, including technical skills and fundamental motor abilities such as strength and speed. Parameters such as maximum contraction force and rate of force development, influence athletic success, although performance gains become harder to achieve as athletic abilities increase. Non-invasive transcranial direct current stimulation of the cerebellum (CB-tDCS) has been used successfully to increase force production in novices, although the potential effects in athletes remain unexplored. The present study examined the effects of CB-tDCS on maximum isometric voluntary contraction force (MVC_iso_) and isometric rate of force development (RFD_iso_) during a bench press task in well-trained athletes. 21 healthy, male, strength-trained athletes participated in a randomized, sham-controlled, double-blinded crossover design. Each participant completed the isometric bench press (iBP) task on two separate days, with at least 5 days between sessions, while receiving either CB-tDCS or sham stimulation. Electromyography (EMG) recordings of three muscles involved in iBP were acquired bilaterally to uncover differences in neuromuscular activation and agonist-antagonist co-contraction between conditions. Contrary to our hypothesis, no significant differences in MVC_iso_ and RFD_iso_ were observed between CB-tDCS and sham conditions. Furthermore, no tDCS-induced differences in neuromuscular activation or agonist-antagonist co-contraction were revealed. Here, we argue that the effects of CB-tDCS on force production appear to depend on the individual's training status. Future research should study individual differences in tDCS responses between athletes and novices, as well as the potential of high-definition tDCS for precise brain region targeting to potentially enhance motor performance in athletic populations.

## 1 Introduction

At the forefront of any athletic development lies the optimization of performance. This involves not only the mastery of sport-specific technical skills but also proficiency in fundamental motor abilities such as strength and speed. Among athletes, the ability to quickly generate high levels of force is crucial to achieving success. Specifically, maximum voluntary contraction force (MVC) and rate of force development (RFD) are key parameters that have been shown to influence athletic performance [1]. While traditional physical training interventions have been successfully applied to improve both MVC and RFD [2], a new branch of potential performance enhancement has emerged in recent years. Non-invasive transcranial direct current stimulation (tDCS) is a method used to modulate brain activity through the application of a weak direct current between surface electrodes placed on an individual's head [3]. Depending on several factors, most notably the polarity of the electrode configuration (anodal or cathodal stimulation), tDCS can induce either inhibition or facilitation of neural excitability. The potential of tDCS as an additional tool to improve performance even in well-trained athletes is based on the concept of "ceiling effects" [4]. This theory posits that performance gains become progressively harder to attain as performance levels increase. Crucially, tDCS may offer a means to overcome these ceiling effects by providing a targeted method to stimulate brain areas involved in motor control and learning [5]. By improving the efficiency of neural processes related to motor performance, tDCS could enhance an athlete's ability to voluntarily generate force and improve peak performance.

Numerous studies have examined the effects of tDCS on strength performance. Particularly in the upper extremities, positive tDCS effects on MVC and other muscle strength parameters are well-documented [[6], [7], [8]]. While lower extremities have also been investigated, results vary regarding its impact. Some findings report tDCS to be effective in modulating muscle strength parameters [[8], [9], [10]], whereas other studies provide evidence to the contrary [[11], [12], [13]]. Many movements are limited, not by MVC, but by the time to develop MVC, making this rate of force development (RFD) another important factor to consider when characterizing muscle strength. RFD mainly relates to motor unit discharge rates [14] and has been shown to increase following a variety of training regimes [[15], [16], [17]] hinting at its positive adaptive responses following sports-related interventions. To our knowledge, only few studies have examined potential tDCS effects on RFD. Cates et al. [18] investigated tDCS effects on RFD in ballistic thumb movements and found an increase in RFD during anodal tDCS over primary motor cortex (M1), while another study reported increased RFD during isolated knee flexion and knee extension, following bilateral M1 stimulation [19].

Notably, most studies within the realm of tDCS-induced strength improvement researched simple or single-joint movements while targeting motor cortical areas, especially M1. In the sporting context, however, compound movements play a particularly important role. Athletic movements typically consist of a combination of simple, mostly single-joint movements that come together to create a compound movement. In the field of strength sports, examples include the deadlift, squat and bench press exercises. Unlike simple movements across a limited number of joints, that primarily engage motor cortical regions such as M1, supplementary motor area, and premotor cortex [20], compound movements require involvement of additional brain areas, particularly the cerebellum [21,22]. The cerebellum is critical concerning motor control of compound movements that necessitate precise and constant motor control [23]. Previous research demonstrated positive effects of cerebellar tDCS (CB-tDCS) on numerous aspects of compound motor performance, e.g., locomotor adaptation [24], postural stability in young [25], and old populations [26], as well as coordinatively-challenging tasks such as adaptive reaching [27] and shooting accuracy [28]. Crucially, only a previous study of our research group investigated the effects of CB-tDCS on muscle strength parameters. Comparing the effectiveness of anodal M1 and anodal CB-tDCS on isometric MVC (MVC_iso_) during a bipedal squat task, we demonstrated a significant increase in MVC_iso_ following anodal CB-tDCS [29]. A mechanistic approach to understanding the connection between CB-tDCS and strength performance hinges on the cerebellum's involvement in agonist-antagonist contractions. Prior research has established a link between favorable adaptations to strength training and decreased antagonistic co-contractions [30]. Equally, individuals with cerebellar pathologies often exhibit deficient antagonistic contraction patterns, manifested by increased co-contractions [31] as well as impaired latencies of agonistic contractions [32]. These specific relationships remained unexplored in our previous study, which is why in this study, we conducted electromyography (EMG) measurements on the agonist-antagonist muscle pair M. triceps brachii and M. biceps brachii to uncover potential differences in agonist-antagonist co-contraction between conditions.

Given that our previous investigation only evaluated untrained individuals, our aim with this study was to expand on our conclusions by examining a well-trained athletic population. As outlined above, this rationale arises from the fact that the effectiveness of tDCS for increasing strength in athletes has not yet been sufficiently explored [33]. Some research has noted modest enhancements in maximal strength during knee extensions [34], while an augmentation in total squat volume has also been observed, albeit without a corresponding increase in maximal strength [35]. Hence, the current evidence remains inadequate to accurately evaluate the extent of validity regarding tDCS-induced strength enhancements. Given the significant variation between athletes and novices in terms of their level of physical performance optimization, it is crucial to include athlete populations in tDCS studies. Gaining insight into potential variations in the mechanisms of tDCS action across different levels of expertise is essential for a comprehensive understanding of tDCS effects. In addition, while the bipedal squat engages the entire body, muscles of the lower extremities are primarily responsible for movement execution. To expand the understanding of tDCS-induced changes in muscle strength parameters during a compound movement of the upper extremities, the present study, therefore, examined changes in MVC_iso_ and isometric RFD (RFD_iso_) during a bench press task. Based on the outlined research, we hypothesized anodal CB-tDCS to increase both MVC_iso_ and RFD_iso_ during iBP compared to sham stimulation (SH-tDCS). Furthermore, we hypothesized a reduction in co-contraction of the biceps brachii for CB-tDCS when compared to SH-tDCS. This hypothesis relates to the effect that anodal CB-tDCS has been shown to increase the inhibitory drive of the cerebellum to M1 [36] and, thereby, potentially downregulates antagonistic co-contraction resulting in increased force production.

Investigating the effects of CB-tDCS on muscle strength parameters in athletes has promising implications for both sports science and broader medical research. Our findings may aid in identifying optimal tDCS protocols for improving muscle strength in athletes, ultimately leading to improved training and competition outcomes. Furthermore, research in this area may provide a deeper understanding of the neural mechanisms underlying muscle strength, which could have implications for the treatment of pathophysiological conditions that affect muscle function.

## 2 Materials and methods

### 2.1 Participants

An a priori power analysis was performed based on previous work from our group showing improvements in MVC_iso_ induced by anodal CB-tDCS during bipedal squats [29]. A power value (probability of correctly rejecting a false null hypothesis) of 0.8 was chosen given a type I error rate α = 5 % and an effect size of 0.48. The estimated minimum sample size to obtain sufficient test power was n = 7. In total, 21 healthy male athletes (aged 25.6 ± 3.7 years (mean ± SD), for detailed overview, please see Table 1) participated in this study. Athletic backgrounds of participants included strength sports, i.e., powerlifting, bodybuilding, strongman (15), football (4) ice-hockey (1) and soccer (1). All participants had no history of neurological disease and no contraindications to tDCS, as determined by a thorough neurological examination. Participants signed an informed consent form in accordance with the Declaration of Helsinki after being informed of the study's aims, procedures, potential risks, and benefits. The study was approved by the ethics committee of Leipzig University (ref.-nr. 034/17-ek).

**Table 1:** Anthropometric and demographic data of the sample (values are expressed as mean ± SD).

Variable	Value
Sample size	n = 21
Age (yrs)	25.6 ± 3.7
Height (cm)	185.1 ± 8.2
Body mass (kg)	92.5 ± 13.5
Strength training experience (yrs)	7.3 ± 4.0
Self-reported bench press maximum (kg)	122.1 ± 35.2
Frequency of bench pressing during typical week	1.8 ± 0.7

### 2.2 Procedures

This experiment consisted of a randomized, sham-controlled, double-blinded cross-over design. Each participant performed a behavioral task of the upper extremities on 2 separate days spaced by at least 5 days to avoid possible impacts of neuromuscular and cognitive fatigue. The behavioral task consisted of standardized isometric bench press (iBP), and all participants were familiar with the bench press exercise through previous strength training experience. On one of the experimental days, anodal CB-tDCS was performed for 20 min. During the second visit, participants received sham stimulation. The order of both experimental days was randomized for each participant.

### 2.3 Isometric bench press (iBP)

**Fig. 1:**
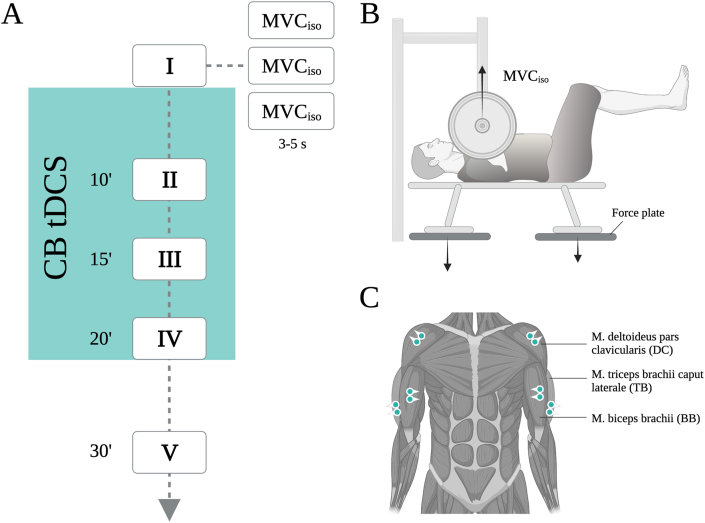
**Experimental Setup. (A)** Each experimental day consisted of five blocks of the isometric bench press (iBP) task, i.e., before (BL1), 10 (BL2), 15 (BL3) and 20 min after tDCS onset (BL4) and 10 min after tDCS termination (BL5). Within each block, three MVC_iso_ were performed for a duration of approximately 5 s each. The duration to complete one block of the iBP task was approximately 1.5 min. tDCS was initiated right after the last MVC_iso_ -measurement of BL1. **(B)** Participants lay on the bench under the barbell at a standardized height and grip width. The legs were lifted off the floor and flexed approximately at right angles at the hip and knee to avoid leg drive during the iBP task. For MVC_iso_-measurements all participants were told to push against the immovable barbell as hard and explosively as possible for 5 s. **(C)** During iBP performance, surface EMG activity of M. deltoideus pars clavicularis (DC), M. triceps brachii caput laterale (TR), and M. biceps brachii (BB) was recorded bilaterally.

Each experimental day consisted of five blocks of the iBP task, i.e., before (BL1), 10 (BL2), 15 (BL3) and 20 min after stimulation onset (BL4), and 10 min after stimulation termination (BL5). Within each block, three MVC_iso_ were performed for a duration of approximately 5 s each (see Fig. 1A). The duration to complete one block of the iBP task was approximately 1.5 min tDCS was initiated right after the last MVC_iso_ -measurement of the first block (BL1).

At the beginning of each experimental session, participants performed a 5 min supervised warm-up consisting of standardized exercises for upper body mobilization and bodyweight pushups. Participants were then placed backwards on a bench to determine individual barbell height and grip width during iBP for standardization of experimental conditions within and between subjects. Both factors were aligned separately for each participant so that the execution of iBP was performed with the arms at a 45° angle to the trunk, the elbows positioned directly under the wrists, and the immovable barbell almost touching the chest at the level of the solar plexus. This was achieved using incrementally adjustable J-hooks within a half-rack. The individual barbell height and grip width were kept constant in both experimental sessions. Before baseline MVC_iso_-measurements were carried out, participants practiced the task for familiarization with submaximal effort.

For the iBP task, participants lay on the bench under the barbell at the specified height and grip width. The legs were lifted off the floor and flexed approximately at right angles at the hip and knee to avoid leg drive during the iBP task (see Fig. 1B). This position was assumed during all measurements. For MVC_iso_-measurement all participants were told to push against the immovable barbell as hard and explosively as possible for 5 s. Per block, three MVC_iso_-measurement (30 s of rest in between each measurement) were averaged and taken for the MVC_iso_-measurement of the respective block. Participants rested in a seated position on the bench in between blocks. During resting phases, movements were prohibited to avoid differences in excitability between participants. No feedback regarding iBP-performance was given during the experiment or between experimental sessions.

### 2.4 Transcranial direct current stimulation

A battery-driven tDCS stimulator (NC-DC-stimulator; neuroConn GmbH, Ilmenau, Germany) was used to deliver current of 2 mA for 20 min (excluding 2 × 30 s of up- and down ramping prior to and after stimulation respectively) by a pair of surface-soaked sponge electrodes. Both tDCS conditions (anodal CB-tDCS, SH-tDCS) were randomly assigned among participants. The anode (35 cm^2^, current density: 0.057 mA/cm^2^) was placed over the bilateral cerebellum, with the cathode (reference; 100 cm^2^, current density 0.020 mA/cm^2^) being placed on the right musculus buccinator [37], both held in place by elastic straps. The anode was placed 2 cm below the inion for anodal cerebellar stimulation [37,38]. During SH-tDCS, a 2 mA current was ramped up for 30 s, maintained for 30 s before being ramped down for 30 s and terminated. Immediately after completion of tDCS, participants were asked to rate the intensity of perceived stimulation on a visual analog scale ranging from 0 (no sensation) to 10 (unbearable sensation).

### 2.5 Data recording

MVC_iso_ (N) was recorded using two multicomponent force plates (Kistler type 9286AA, Kistler AG, Winterthur, Switzerland) using a sampling rate of 1500Hz. During the iBP performance, the force plates were positioned directly underneath the bench that participants were laying on, which was placed inside a half-rack (Barbarian-Line® Profi Half Rack, IFS GmbH, Wassenberg, Germany, see Fig. 1B).

During iBP performance, muscle activity of M. deltoideus pars clavicularis (DC), M. triceps brachii caput laterale (TR), and M. biceps brachii (BB) was recorded bilaterally (see Fig. 1C) using a wireless Desktop Transmission System (NORAXON Inc., Scottsdale, AZ). DC and TR were assessed to investigate the influence of CB-tDCS on muscle activation of prime movers of iBP. The position of participants during iBP (barbell almost touching the chest) corresponds roughly to the sticking period of the bench press, where prime movers such as DC and TR have been shown to exhibit high muscle activation [39]. BB was additionally recorded to investigate the influence of CB-tDCS on agonist-antagonist co-contraction (TR/BB). Optimal signal quality during recording was assured through skin preparation, i.e., shaving, abrasion, and cleaning with alcohol. Next, gel-coated self-adhesive surface electrodes (interelectrode distance of 20 mm) were mounted on standardized electrode positions according to SENIAM recommendations [40]. Based on anatomical landmarks, all electrode placements were kept constant between sessions. EMG electrodes were positioned pairwise in parallel to the directions of the muscle fibers. The sampling rate used for the data collection was 1500 Hz, the input impedance of the amplifier was set at >100 MΩ, bandpass filtering was applied in the frequency range of 10–500 Hz, and common-mode rejection (CMRR) was set at >100 dB. Furthermore, maximum voluntary contraction (MVC) values were determined for each muscle at the beginning of every single experimental session to enable normalization of EMG activity. To determine the MVC of DC, TR, and BB, three maximal isometric contractions (5 s) of each muscle were performed in standardized positions. Amplitude normalization of all trials in a testing session was carried out using the maximum RMS value of all three MVCs of each participant for each muscle separately. Between each MVC trial, a 30-s rest period was granted.

### 2.6 Data analysis

Data analyses were performed using customized MATLAB® scripts (v. R2023a, The MathWorks Inc., Natick, USA).

**Fig. 2:**
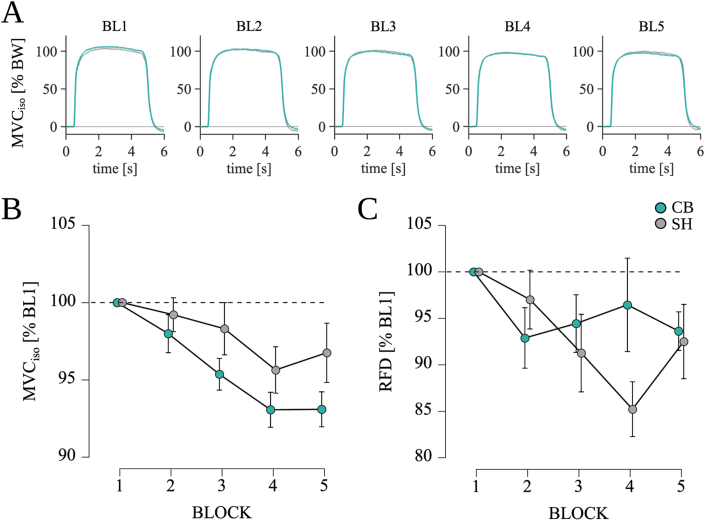
**Isometric bench press (iBP) performance. (A)** Mean force data of all participants normalized to the individual bodyweight for CB-tDCS and SH-tDCS. **(B)** MVC_iso_ showed no significant difference between CB-tDCS and SH-tDCS. **(C)** No significant differences between CB-tDCS and SH-tDCS regarding rate of force development (RFD). Indicated are mean values, the error bars represent the SEM.

Individual force data were evaluated thoroughly with incorrect measurements being excluded. Force values (N), corresponding to the average of all values exceeding the 95th quantile, were extracted out of three measurements for each block and averaged to make for a robust MVC_Iso_ value (cf., Figs. 1A and 2A). Data were then normalized with respect to individual baseline performances (BL1) on each different session (CB-tDCS, SH-tDCS).

RFD values (N^**.**^s^−1^) are defined as the steepest slope of the force-time curve according to Aagaard et al. [41]. Force-time curve onsets were defined manually by a single trained researcher. RFD values were extracted for each of the three iBP-measurements of each block and subsequently averaged. Mean RFD values for each block were normalized to baseline values (BL1) for each experimental session separately and used for further analyses.

EMG amplitudes of DC, TR and BB were computed from the EMG signal recorded during each iBP trial. Therefore, muscle on- and offsets were visually determined by a single trained researcher. Next, RMS values were obtained and mean amplitudes were calculated. Before further analysis, all EMG amplitudes were normalized to individual MVC values recorded at the beginning of each experimental session. Lastly, MVC normalized EMG amplitudes were averaged for each block before statistical analysis.

All data were normally distributed as assessed through Lilliefors-testing (α = 0.05). Baseline values (BL1) of raw MVC_iso_ (in N) were compared between conditions (CB-tDCS, SH-tDCS) using a paired samples *t*-test. Subsequently, normalized MVC_iso_ for each block were subjected to repeated-measures ANOVA with the within-subject factors STIM (CB-tDCS, SH-tDCS) and TIME (BL1-BL5) to test for influences of tDCS on iBP performance. The same analysis was used to separately assess influences of tDCS on RFD. MVC normalized EMG amplitudes were analyzed by a separate repeated measures ANOVA with the within-subject factors STIM (CB-tDCS, SH-tDCS), TIME (BL1-BL5), SIDE (RIGHT, LEFT) and MUSCLE (DC, TR, BB). In order to assess agonist-antagonist co-contraction during iBP performance, the EMG ratio between TR and BB amplitudes was computed blockwise for each day separately (TR/BB). Therefore, bilateral EMG amplitudes of the same muscle were averaged, i.e., left and right TR, for each day and block. Finally, a repeated-measures ANOVA with the within-subject factors STIM (CB-tDCS, SH-tDCS) and TIME (BL1-BL5) was performed to test for tDCS-induced modulations of TR/BB. Potential sphericity violations were corrected with Greenhouse Geisser correction. Statistical thresholds were set at p < 0.05. Post hoc analyses were conducted with Bonferroni correction for multiple comparisons.

Lastly, Spearman rank correlation was used to test for associations between self-reported bench press maximum weight and initial iBP performance (BL1) on each day separately. Furthermore, Spearman rank correlation was performed between initial iBP performance (BL1) and the percentage difference between BL1 and BL5 (ΔMVC_iso_) on each day separately. To assess relationships between RFD and underlying muscle activation, Spearman rank correlations between RFD values and bilaterally averaged EMG amplitudes were computed blockwise for each muscle and day separately.

## 3 Results

Perceived sensation of tDCS showed no difference between CB-tDCS and SH-tDCS (mean difference (MD) = 0.52, t_(20)_ = 1.206, p = 0.242, d = 0.263), indicating the effectiveness of blinding procedures.

### 3.1 Isometric bench press performance

Baseline performance (BL1) of MVC_iso_ did not differ between CB-tDCS and SH-tDCS stimulation sessions (MD = 23.58 N, t_(20)_ = 1.435, p = 0.167, d = 0.313).

Repeated measures ANOVA revealed a significant effect for TIME on MVC_iso_ (F_(2.94, 58.80)_ = 8.019, p < 0.001, η_p_^2^ = 0.286, see Fig. 2B). Pairwise post-hoc testing indicated a decrease of MVC_iso_ from BL1 to BL4 (MD = −5.65 %, SE = 1.2 %, p < 0.001, d = −0.800), BL1 to BL5 (MD = −5.08 %, SE = 1.2 %, p < 0.001, d = −0.718), BL2 to BL4 (MD = −4.26 %, SE = 1.2 %, p = 0.005, d = −0.602) and BL2 to BL5 (MD = −3.68 %, SE = 1.2 %, p = 0.020, d = −0.521). However, no significant effect of STIM (F_(1, 20)_ = 1.932, p = 0.180, η_p_^2^ = 0.088) and no significant interaction STIM*TIME (F_(4, 20)_ = 1.067, p = 0.379, η_p_^2^ = 0.051) was found.

Regarding RFD, a significant effect for TIME (F_(4, 20)_ = 2.527, p = 0.047, η_p_^2^ = 0.112) was revealed (see Fig. 2C). Pairwise post-hoc testing indicated a decrease of RFD from BL1 to BL4 (MD = −9.17 N s^−1^, SE = 3.1 N s^−1^, p = 0.041, d = −0.500). Again, no significant effect for STIM (F_(1, 20)_ = 0.357, p = 0.557, η_p_^2^ = 0.018) or interaction STIM*TIME (F_(2.66, 20)_ = 2.283, p = 0.097, η_p_^2^ = 0.102) could be observed.

### 3.2 EMG data

**Fig. 3:**
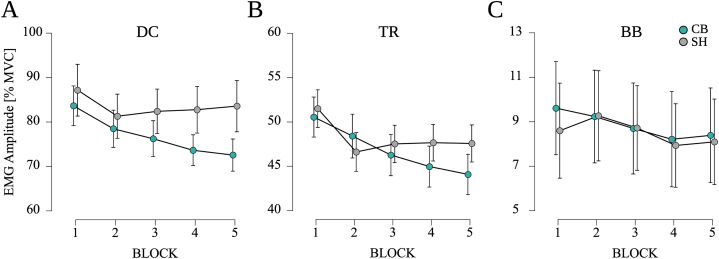
**Mean EMG amplitudes for (A)** M. deltoideus pars clavicularis (DC), **(B)** M. triceps brachii (TR) and **(C)** M. biceps brachii (BB). No significant differences in muscle activation were observed between CB-tDCS and SH-tDCS. Indicated are mean values, the error bars represent the SEM.

With respect to MVC normalized EMG amplitudes, significant effects for MUSCLE (F_(1.55, 29.46)_ = 103.320, p < 0.001, η_p_^2^ = 0.845) and TIME (F_(2.55, 29.46)_ = 16.322, p < 0.001, η_p_^2^ = 0.477) were observed (see Fig. 3A–C). Pairwise post-hoc testing revealed higher EMG amplitudes for DC compared to TR (MD = 32.7 %, SE = 5.0 %, p < 0.001, d = 1.469) and BB (MD = 71.5 %, SE = 5.0 %, p < 0.001, d = 3.215) as well as higher EMG amplitudes for TR compared to BB (MD = 38.8 %, SE = 5.0 %, p < 0.001, d = 1.746). Furthermore, EMG amplitudes were higher during BL1 compared to BL2 (MD = 3.0 %, SE = 0.6 %, p < 0.001, d = 0.133), BL3 (MD = 3.5 %, SE = 0.6 %, p < 0.001, d = 0.159), BL4 (MD = 4.3 %, SE = 0.6 %, p < 0.001, d = 0.194) and BL5 (MD = 4.5 %, SE = 0.6 %, p < 0.001, d = 0.201). However, no significant effect for STIM (F_(1, 38)_ = 0.809, p = 0.380, η_p_^2^ = 0.041), SIDE (F_(1, 38)_ = 3.430, p = 0.080, η_p_^2^ = 0.153) and no interaction effect STIM*MUSCLE (F_(1, 19)_ = 0.123, p = 0.730) and STIM*TIME (F_(1, 19)_ = 1.859, p = 0.170) were found.

Regarding agonist-antagonist co-contraction (TR/BB), no significant effect for STIM (F_(1, 19)_ = 0.993, p = 0.331, η_p_^2^ = 0.050), TIME (F_(2.81, 53.33)_ = 1.404, p = 0.253, η_p_^2^ = 0.069) and no interaction effect STIM*TIME (F_(2.23, 19)_ = 0.975, p = 0.394, η_p_^2^ = 0.049) was found.

### 3.3 Correlation analysis

Spearman rank correlation revealed highly significant associations between self-reported bench press maximum weight and initial performance during CB-tDCS (r_s_ = 0.842, p < 0.001, z = 1.228) and SH-tDCS (r_s_ = 0.827, p < 0.001, z = 1.179).

**Fig. 4:**
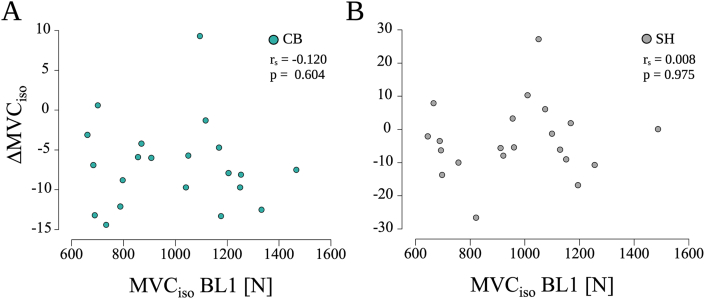
**Spearman rank correlation between initial iBP performance (BL1) and percentage change in performance from BL1 to BL5 (ΔMVC**_**iso**_**). (A)** CB-tDCS, **(B)** SH-tDCS. No significant associations were found during CB-tDCS or SH-tDCS.

However, no significant correlation between initial iBP performance and ΔMVC_iso_ was found for CB-tDCS (r_s_ = −0.120, p = 0.604, z = −0.122, see Fig. 4A) and SH-tDCS (r_s_ = 0.008, p = 0.975, z = 0.008, see Fig. 4B).

Furthermore, no significant correlations between RFD values and muscle EMG amplitudes were found for CB-tDCS (all p > 0.229) and SH-tDCS (all p > 0.123).

## 4 Discussion

With the present study, we aimed to investigate the effects of anodal CB-tDCS on MVC_iso_ and RFD_iso_ during an isometric bench press task. We did not observe significant differences in MVC_iso_ or RFD_iso_ between anodal CB-tDCS and SH-tDCS conditions. Finally, we were unable to observe differences in neuromuscular activation or agonist-antagonist co-contraction between CB-tDCS and SH-tDCS. These findings contrast with a previous study of our group [29], where significant improvements in MVC_iso_ were demonstrated in novices performing an isometric squat task following anodal CB-tDCS. These discrepant outcomes suggest that the impact of cerebellar tDCS on force production exhibits a multifaceted nature, depending on various factors, including an individual's level of training, and experience, among other relevant aspects which will be discussed in detail in the following.

The modulatory capacity of tDCS, specifically in the domain of motor performance, is known to be heterogenous. In recent years, there have been reports presenting evidence both supporting and opposing a potentially positive impact of tDCS administration with different stimulation protocols and target areas on motor performance [42]. Several studies demonstrated force-enhancing effects of tDCS, while others were unable to reproduce such findings [43]. In general, the effects of tDCS on force parameters demonstrate considerable variability, particularly when addressing conditions with high force and/or velocity demands. For example, Alix-Fages et al. [44] observed negligible impacts on maximal muscle force, velocity, and power, indicating that tDCS over the DLPFC may not enhance non-fatigued sprint performance. Another study by the same authors found no improvements in running performance or perceived exertion during repeated sprint ability tasks with either anodal or cathodal tDCS over the DLPFC [45]. Romero-Arenas et al. [46] found neither anodal nor cathodal tDCS over the DLPFC to enhance countermovement jump performance, while Kristiansen et al. [47] noted no significant changes in corticospinal excitability or maximal voluntary isometric contraction force after tDCS applied over M1, suggesting limited potential for enhancing quadriceps muscle strength in healthy individuals.

Importantly, the prevailing force-enhancing tDCS protocols primarily involved the application of anodal tDCS targeting motor or prefrontal brain regions. Acknowledging the significant role of the cerebellum in compound motor control, we extended previous approaches by effectively utilizing CB-tDCS to enhance isometric force output during a whole-body movement [29]. In order to enhance our understanding of the force enhancement induced by CB-tDCS and evaluate the broader applicability of this method, we expanded our experimental approach in two significant ways. First, we examined trained athletes to evaluate the efficacy of CB-tDCS to enhance force production in a group of experts. Second, we examined iBP, a compound movement involving the upper extremities, to broaden the scope of our previous study which solely focused on a compound movement of the lower extremities.

A primary rationale for the lack of force enhancement observed in this study, centers on the role of the cerebellum in compound motor control. The cerebellum plays a critical role in motor coordination and motor learning, and its stimulation through tDCS can modulate neural activity and plasticity [48], although this has not been irrevocably established [49]. The neural mechanisms underlying force production during bench pressing and squatting may involve distinct neural networks and motor control pathways. Evidence in favor of limb or muscle-specific motor performance modulation following anodal CB-tDCS comes from studies showing selective performance enhancement between movements of the hands and arms during visuomotor adaptation tasks [50]. Here, visuomotor adaptation was improved only for movements of the arm following anodal CB-tDCS. Interestingly, an inverted effect has been observed for cathodal cerebellar stimulation [51]. Recent meta-analyses on post-stroke recovery showed differential modulations for upper and lower extremities following tDCS administration further highlighting potentially distinct neural interplay for muscles of the upper and lower body [52]. To gain initial insight into differences in neuromuscular processing between CB-tDCS and SH-tDCS, we investigated surface EMG amplitudes of three muscles engaged in iBP. Analysis of EMG amplitudes did not reveal significant differences between CB-tDCS and SH-tDCS. The absence of notable differences in EMG amplitude between CB-tDCS and SH-tDCS conditions is probably because CB-tDCS did not adequately affect neuromuscular parameters. Previous research has noted EMG amplitude increases corresponding to higher force levels, aligning with the primarily linear relationship between EMG amplitudes and force [19]. Since we failed to observe differences in strength and EMG amplitudes between conditions, we, therefore, argue that CB-tDCS did not selectively alter muscle activation profiles during iBP, although this reasoning is limited to the muscles analyzed in this study and may not apply to additional muscles involved in iBP. Similarly, we did not detect any alterations in the agonist-antagonist co-contraction between TR and BB when comparing CB-tDCS to SH-tDCS. This finding contrasts our initial hypothesis. Given the cerebellum's integral role in governing these modes of muscle contraction, we anticipated a distinct influence of CB-tDCS. This lack of effect could be attributed to the broad-scale stimulation of the cerebellum in the present study. Preliminary research into the cerebellum's role in agonist-antagonist contractions has revealed that impaired contraction patterns arise from the inactivation of particular cerebellar nuclei, specifically the interposed and dentate nuclei [53]. Consequently, stimulating the entire cerebellum may lack the specificity required to induce the desired optimized contraction patterns. Future studies should focus on the role of agonist-antagonist co-contraction following CB-tDCS-induced motor performance enhancements by increasing the focality and tailoring other important stimulation parameters of CB-tDCS. Additionally, explicitly accessing cerebellar inhibitory drive to motor output regions such as M1 should be investigated in future studies via the use of non-invasive brain stimulation protocols such as paired-pulse TMS.

To gain a comprehensive understanding of the neural processes underlying CB-tDCS induced force modulation, it is crucial to explore concurrent analyses examining both brain and muscle activity during task performance. By integrating these complementary analyses, we may progress to uncovering the intricate interplay between the brain and muscles, thereby advancing our understanding of the effects of CB-tDCS on force modulation.

All participants in the current study were trained athletes, whereas our previous study involved novice individuals. Athletes already possess a higher level of motor skill and proficiency in executing specific movements compared to novices. The potential for further improvement in force production may be limited in trained athletes due to their already optimized motor performance [5,54]. In contrast, novice individuals likely exhibit greater potential for improvement as they undergo rapid skill acquisition during the early stages of motor learning [55]. Consequently, several studies that effectively utilized anodal CB-tDCS to augment motor performance focused on novel motor tasks with a considerable potential for improvement [[56], [57], [58], [59]]. This sentiment is further supported by studies that specifically investigated athletes or individuals with substantial experience in the task under study. For example, Mizuguchi et al. [60] conducted a study to examine the impact of CB-tDCS administration on dart-throwing performance. Interestingly, they observed a significant improvement only in participants with initially low performance following cathodal CB-tDCS, whereas anodal CB-tDCS did not yield the same effect. Contrary to these findings, Kamali et al. [28] successfully improved shooting performance of trained pistol shooters following anodal CB-tDCS. Critically, the authors used a multi-modal stimulation protocol, where activity of the dorsolateral prefrontal cortex (dlPFC) was additionally suppressed. In another study, trained gymnasts were investigated to evaluate the effects of bilateral anodal CB-tDCS on static and dynamic strength performance [61]. Again, no performance improvements were observed for static and dynamic strength in the study. Expanding on this notion, the training status of the participants could be a crucial factor influencing the response to cerebellar tDCS. Trained athletes typically undergo specific strength training regimens tailored to their sport, leading to specific adaptations in the central nervous system and muscle function [62]. These adaptations might render the athletes less responsive to the effects of tDCS, as their neuromuscular systems are already optimized through years of training. Notably, all participants included in this study consistently incorporated the bench press exercise into their training regimens (cf. Table 1). Since previous research has demonstrated that the efficacy of tDCS can be influenced by initial performance level and athletic background, we conducted a correlational analysis between initial MVC_iso_ and ΔMVC_iso_ to investigate a potential association between initial performance and performance improvement. However, we were unable to replicate such a relationship. It is worth noting that Mizuguchi et al. [60] observed a similar link only for cathodal CB-tDCS and, consistent with our findings, did not find a correlation for anodal CB-tDCS. The authors considered that cathodal CB-tDCS may reduce neural noise within the cerebellum, thereby facilitating motor learning in the subgroup with lower performance. It is tempting to speculate that such a mechanism might also facilitate force enhancement, although further research utilizing cathodal CB-tDCS is needed to address this question.

### 4.1 Limitations

While the current study was intentionally designed to build upon a previous study conducted by our group, it is important to acknowledge and address certain limitations. In order to enhance the validity of MVC measurements, we modified the number of MVC tests per MVC block, increasing it from one measurement to three measurements. However, this adjustment also introduced fatigue as a contributing factor, which potentially led to a decrease in MVC for both CB-tDCS and SH-tDCS. Although both conditions resulted in similar fatigue levels, it is plausible that the accumulation of fatigue masked potential performance-enhancing effects of tDCS stimulation. Nevertheless, prior research by Ref. [63] indicates a positive impact of tDCS on fatiguability, albeit only in the context of cortical tDCS configurations. Another limitation stems from the broad target area used in our study. The tDCS configuration employed here was designed to stimulate the bilateral cerebellum extensively. While such broad stimulation may be sufficient to enhance performance in novices, it may not be optimal for athletes who could require more localized stimulation. This perspective is supported by research indicating that only specific regions of the cerebellum are associated with force production parameters such as amplitude and RFD [64]. Consequently, our stimulation may have encompassed too wide an area, limiting its ability to induce specific force production-related effects. To address this issue, future studies should explore the use of high-definition tDCS (HD-tDCS), which allows for more focused targeting of specific areas. Finally, we chose to include only male participants in this study to avoid potential confounding effects resulting from sex-related differences in central nervous system structure and function [65]. Therefore, our findings are specific to males and cannot be generalized to female populations. Future research should explore an all-female group to better understand the broader implications of our results.

### 4.2 Conclusion

In conclusion, this study contributes to the growing understanding of the complex effects of CB-tDCS on force production. Further investigation is needed to uncover the underlying neural mechanisms, the influence of specific motor tasks, and the role of individual differences in response to CB-tDCS, specifically in athletic populations. The cumulative findings derived from such research have the potential to advance the development of optimized tDCS protocols aimed at enhancing motor performance not only in athletes but also in various other populations.

## Data availability statement

Data, in an anonymous format (according to data protection policy in the ethics agreement), is available at https://doi.org/10.6084/m9.figshare.23708496.v1.

## Funding

No funding was received for conducting this study.

## CRediT authorship contribution statement

**Rouven Kenville:** Writing – review & editing, Writing – original draft, Visualization, Methodology, Investigation, Formal analysis, Data curation, Conceptualization. **Clauß Martina:** Writing – review & editing, Formal analysis, Data curation. **Stefan Berkow:** Writing – review & editing, Data curation. **Patrick Ragert:** Writing – review & editing, Supervision. **Tom Maudrich:** Writing – review & editing, Writing – original draft, Visualization, Methodology, Investigation, Formal analysis, Data curation, Conceptualization.

## Declaration of competing interest

The authors declare that they have no known competing financial interests or personal relationships that could have appeared to influence the work reported in this paper.

## References

[1] Maffiuletti N.A., Aagaard P., Blazevich A.J., Folland J., Tillin N., Duchateau J. (2016). Rate of force development: physiological and methodological considerations. Eur. J. Appl. Physiol..

[2] Aagaard P., Simonsen E., Andersen J., Magnusson P., Dyhre-Poulsen P. (2002). Increased rate of force development and neural drive of human skeletal muscle following resistance training. J. Appl. Physiol..

[3] Nitsche M.A., Paulus W. (2000). Excitability changes induced in the human motor cortex by weak transcranial direct current stimulation. J. Physiol..

[4] Wernbom M., Augustsson J., Thomee R. (2007). The influence of frequency, intensity, volume and mode of strength training on whole muscle cross-sectional area in humans. Sports Med..

[5] da Silva Machado D.G., Bikson M., Datta A., Caparelli-Dáquer E., Unal G., Baptista A.F., Cyrino E.S., Li L.M., Morya E., Moreira A., Okano A.H. (2021). Acute effect of high-definition and conventional tDCS on exercise performance and psychophysiological responses in endurance athletes: a randomized controlled trial. Sci. Rep..

[6] Abdelmoula A., Baudry S., Duchateau J. (2016). Anodal transcranial direct current stimulation enhances time to task failure of a submaximal contraction of elbow flexors without changing corticospinal excitability. Neuroscience.

[7] Vargas V.Z., Baptista A.F., Pereira G.O.C., Pochini A.C., Ejnisman B., Santos M.B., Joao S.M.A., Hazime F.A. (2018). Modulation of isometric quadriceps strength in soccer players with transcranial direct current stimulation: a crossover study. J. Strength Condit Res..

[8] Tanaka S., Hanakawa T., Honda M., Watanabe K. (2009). Enhancement of pinch force in the lower leg by anodal transcranial direct current stimulation. Exp. Brain Res..

[9] Angius L., Pageaux B., Hopker J., Marcora S.M., Mauger A.R. (2016). Transcranial direct current stimulation improves isometric time to exhaustion of the knee extensors. Neuroscience.

[10] Lattari E., Rosa Filho B.J., Fonseca Junior S.J., Murillo-Rodriguez E., Rocha N., Machado S., Maranhao Neto G.A. (2018). Effects on volume load and ratings of perceived exertion in individuals advanced weight-training after transcranial direct current stimulation. J. Strength Condit Res..

[11] Lattari E., Campos C., Lamego M.K., Passos de Souza S.L., Neto G.M., Rocha N.B., Jose de Oliveira A., Carpenter S., Machado S. (2017). Can transcranial direct current stimulation improve muscle power in individuals with advanced resistance training experience?. J. Strength Condit Res..

[12] Ciccone A.B., Deckert J.A., Schlabs C.R., Tilden M.J., Herda T.J., Gallagher P.M., Weir J.P. (2018). Transcranial direct current stimulation of the temporal lobe does not affect high intensity work capacity. J. Strength Condit Res..

[13] Maeda K., Yamaguchi T., Tatemoto T., Kondo K., Otaka Y., Tanaka S. (2017). Transcranial direct current stimulation does not affect lower extremity muscle strength training in healthy individuals: a triple-blind, sham-controlled study. Front. Neurosci..

[14] Duchateau J., Baudry S. (2014). Maximal discharge rate of motor units determines the maximal rate of force development during ballistic contractions in human. Front. Hum. Neurosci..

[15] Blazevich A.J., Cannavan D., Horne S., Coleman D.R., Aagaard P. (2009). Changes in muscle force–length properties affect the early rise of force in vivo. Muscle Nerve.

[16] Blazevich A.J., Horne S., Cannavan D., Coleman D.R., Aagaard P. (2008). Effect of contraction mode of slow‐speed resistance training on the maximum rate of force development in the human quadriceps. Muscle Nerve: Official Journal of the American Association of Electrodiagnostic Medicine.

[17] Hernández-Davó J.L., Sabido R. (2014). Rate of force development: reliability, improvements and influence on performance. A review. European Journal of Human Movement.

[18] Cates A., Lin R., Wingeier B. (2019). Abstract# 70: effect of tDCS over motor cortex on isometric rate of force development in healthy adults. Brain Stimul.: Basic, Translational, and Clinical Research in Neuromodulation.

[19] Lu P., Hanson N.J., Wen L., Guo F., Tian X. (2021). Transcranial direct current stimulation enhances muscle strength of non-dominant knee in healthy young males. Front. Physiol..

[20] Cheney P.D. (1985). Role of cerebral cortex in voluntary movements. A review. Phys. Ther..

[21] Ioffe M.E., Chernikova L.A., Ustinova K.I. (2007). Role of cerebellum in learning postural tasks. Cerebellum.

[22] Thach W.T. (1975). Timing of activity in cerebellar dentate nucleus and cerebral motor cortex during prompt volitional movement. Brain Res..

[23] Jacobs J.V., Horak F.B. (2007). Cortical control of postural responses. J. Neural. Transm..

[24] Jayaram G., Tang B., Pallegadda R., Vasudevan E.V., Celnik P., Bastian A. (2012). Modulating locomotor adaptation with cerebellar stimulation. J. Neurophysiol..

[25] Poortvliet P., Hsieh B., Cresswell A., Au J., Meinzer M. (2018). Cerebellar transcranial direct current stimulation improves adaptive postural control. Clin. Neurophysiol..

[26] Yosephi M.H., Ehsani F., Zoghi M., Jaberzadeh S. (2018). Multi-session anodal tDCS enhances the effects of postural training on balance and postural stability in older adults with high fall risk: primary motor cortex versus cerebellar stimulation. Brain Stimul..

[27] Hardwick R.M., Celnik P.A. (2014). Cerebellar direct current stimulation enhances motor learning in older adults. Neurobiol. Aging.

[28] Kamali A.M., Nami M., Yahyavi S.S., Saadi Z.K., Mohammadi A. (2019). Transcranial direct current stimulation to assist experienced pistol shooters in gaining even-better performance scores. Cerebellum.

[29] Kenville R., Maudrich T., Maudrich D., Villringer A., Ragert P. (2020). Cerebellar transcranial direct current stimulation improves maximum isometric force production during isometric barbell squats. Brain Sci..

[30] Carolan B., Cafarelli E. (1992). Adaptations in coactivation after isometric resistance training. J. Appl. Physiol..

[31] Hallett M., Shahani B.T., Young R.R. (1975). EMG analysis of patients with cerebellar deficits. J. Neurol. Neurosurg. Psychiatr..

[32] Diener H.C., Dichgans J. (1992). Pathophysiology of cerebellar ataxia. Mov. Disord.: official journal of the Movement Disorder Society.

[33] Maudrich T., Ragert P., Perrey S., Kenville R. (2022). Single-session anodal transcranial direct current stimulation to enhance sport-specific performance in athletes: a systematic review and meta-analysis. Brain Stimul..

[34] Kamali A.M., Saadi Z.K., Yahyavi S.S., Zarifkar A., Aligholi H., Nami M. (2019). Transcranial direct current stimulation to enhance athletic performance outcome in experienced bodybuilders. PLoS One.

[35] Fortes L.S., Mazini-Filho M., Lima-Júnior D., Machado D.G.S., Albuquerque M.R., Fonseca F.S., Ferreira M.E.C. (2021). Transcranial stimulation improves volume and perceived exertion but does not change power. Int. J. Sports Med..

[36] Galea J.M., Jayaram G., Ajagbe L., Celnik P. (2009). Modulation of cerebellar excitability by polarity-specific noninvasive direct current stimulation. J. Neurosci..

[37] Ferrucci R., Cortese F., Priori A. (2015). Cerebellar tDCS: how to do it. Cerebellum.

[38] Taubert M., Stein T., Kreutzberg T., Stockinger C., Hecker L., Focke A., Ragert P., Villringer A., Pleger B. (2016). Remote effects of non-invasive cerebellar stimulation on error processing in motor Re-learning. Brain Stimul..

[39] Van Den Tillaar R., Ettema G. (2010). The “sticking period” in a maximum bench press. J. Sports Sci..

[40] Hermens H.J., Freriks B., Disselhorst-Klug C., Rau G. (2000). Development of recommendations for SEMG sensors and sensor placement procedures. J. Electromyogr. Kinesiol..

[41] Aagaard P., Simonsen E.B., Andersen J.L., Magnusson P., Dyhre-Poulsen P. (2002). Increased rate of force development and neural drive of human skeletal muscle following resistance training. J. Appl. Physiol..

[42] Reis J., Fritsch B. (2011). Modulation of motor performance and motor learning by transcranial direct current stimulation. Curr. Opin. Neurol..

[43] Alix-Fages C., Romero-Arenas S., Castro-Alonso M., Colomer-Poveda D., Río-Rodriguez D., Jerez-Martínez A., Fernandez-del-Olmo M., Márquez G. (2019). Short-term effects of anodal transcranial direct current stimulation on endurance and maximal force production: a systematic review and meta-analysis. J. Clin. Med..

[44] Alix-Fages C., Garcia-Ramos A., Romero-Arenas S., Nadal G.C., Jerez-Martinez A., Colomer-Poveda D., Marquez G. (2022). Transcranial direct current stimulation does not affect sprint performance or the horizontal force-velocity profile. Res. Q. Exerc. Sport.

[45] Alix-Fages C., Romero-Arenas S., Calderon-Nadal G., Jerez-Martinez A., Pareja-Blanco F., Colomer-Poveda D., Marquez G., Garcia-Ramos A. (2022). Transcranial direct current stimulation and repeated sprint ability: No effect on sprint performance or ratings of perceived exertion. Eur. J. Sport Sci..

[46] Romero-Arenas S., Calderon-Nadal G., Alix-Fages C., Jerez-Martinez A., Colomer-Poveda D., Marquez G. (2021). Transcranial direct current stimulation does not improve countermovement jump performance in young healthy men. J. Strength Condit Res..

[47] Kristiansen M., Thomsen M.J., Norgaard J., Aaes J., Knudsen D., Voigt M. (2022). The effect of anodal transcranial direct current stimulation on quadriceps maximal voluntary contraction, corticospinal excitability, and voluntary activation levels. J. Strength Condit Res..

[48] Kumari N., Taylor D., Signal N. (2019). The effect of cerebellar transcranial direct current stimulation on motor learning: a systematic review of randomized controlled trials. Front. Hum. Neurosci..

[49] Jalali R., Miall R.C., Galea J.M. (2017). No consistent effect of cerebellar transcranial direct current stimulation on visuomotor adaptation. J. Neurophysiol..

[50] Weightman M., Brittain J.S., Punt D., Miall R.C., Jenkinson N. (2020). Targeted tDCS selectively improves motor adaptation with the proximal and distal upper limb. Brain Stimul..

[51] Weightman M., Brittain J.S., Miall R.C., Jenkinson N. (2021). Direct and indirect effects of cathodal cerebellar TDCS on visuomotor adaptation of hand and arm movements. Sci. Rep..

[52] Bai X., Guo Z., He L., Ren L., McClure M.A., Mu Q. (2019). Different therapeutic effects of transcranial direct current stimulation on upper and lower limb recovery of stroke patients with motor dysfunction: a meta-analysis. Neural Plast..

[53] Vilis T., Hore J. (1977). Effects of changes in mechanical state of limb on cerebellar intention tremor. J. Neurophysiol..

[54] Hummel F.C., Voller B., Celnik P., Floel A., Giraux P., Gerloff C., Cohen L.G. (2006). Effects of brain polarization on reaction times and pinch force in chronic stroke. BMC Neurosci..

[55] Kan B., Dundas J.E., Nosaka K. (2013). Effect of transcranial direct current stimulation on elbow flexor maximal voluntary isometric strength and endurance. Appl. Physiol. Nutr. Metabol..

[56] Cantarero G., Spampinato D., Reis J., Ajagbe L., Thompson T., Kulkarni K., Celnik P. (2015). Cerebellar direct current stimulation enhances on-line motor skill acquisition through an effect on accuracy. J. Neurosci..

[57] Grami F., de Marco G., Bodranghien F., Manto M., Habas C. (2022). Cerebellar transcranial direct current stimulation reconfigures brain networks involved in motor execution and mental imagery. Cerebellum.

[58] Jackson A.K., de Albuquerque L.L., Pantovic M., Fischer K.M., Guadagnoli M.A., Riley Z.A., Poston B. (2019). Cerebellar transcranial direct current stimulation enhances motor learning in a complex overhand throwing task. Cerebellum.

[59] Lindberg P.G., Verneau M., Boterff Q.L., Cuenca-Maia M., Baron J.C., Maier M.A. (2022). Age- and task-dependent effects of cerebellar tDCS on manual dexterity and motor learning-A preliminary study. Neurophysiol. Clin..

[60] Mizuguchi N., Katayama T., Kanosue K. (2018). The effect of cerebellar transcranial direct current stimulation on A throwing task depends on individual level of task performance. Neuroscience.

[61] Anoushiravani S., Alizadehgoradel J., Iranpour A., Yousefi Bilehsavar O., Pouresmali A., Nitsche M.A., Salehinejad M.A., Mosayebi-Samani M., Zoghi M. (2023). The impact of bilateral anodal transcranial direct current stimulation of the premotor and cerebellar cortices on physiological and performance parameters of gymnastic athletes: a randomized, cross-over, sham-controlled study. Sci. Rep..

[62] Yarrow K., Brown P., Krakauer J.W. (2009). Inside the brain of an elite athlete: the neural processes that support high achievement in sports. Nat. Rev. Neurosci..

[63] Lattari E., Oliveira B.R.R., Monteiro Junior R.S., Marques Neto S.R., Oliveira A.J., Maranhao Neto G.A., Machado S., Budde H. (2018). Acute effects of single dose transcranial direct current stimulation on muscle strength: a systematic review and meta-analysis. PLoS One.

[64] Spraker M.B., Corcos D.M., Kurani A.S., Prodoehl J., Swinnen S.P., Vaillancourt D.E. (2012). Specific cerebellar regions are related to force amplitude and rate of force development. Neuroimage.

[65] Grabowska A. (2017). Sex on the brain: are gender‐dependent structural and functional differences associated with behavior?. J. Neurosci. Res..

